# A high density physical map of chromosome 1BL supports evolutionary studies, map-based cloning and sequencing in wheat

**DOI:** 10.1186/gb-2013-14-6-r64

**Published:** 2013-06-25

**Authors:** Romain Philippe, Etienne Paux, Isabelle Bertin, Pierre Sourdille, Fréderic Choulet, Christel Laugier, Hana Šimková, Jan Šafář, Arnaud Bellec, Sonia Vautrin, Zeev Frenkel, Federica Cattonaro, Federica Magni, Simone Scalabrin, Mihaela M Martis, Klaus FX Mayer, Abraham Korol, Hélène Bergès, Jaroslav Doležel, Catherine Feuillet

**Affiliations:** 1 INRA-UBP UMR 1095 Genetics, Diversity and Ecophysiology of Cereals, 5 Chemin de Beaulieu 63039 Clermont-Ferrand, France; 2 Centre of the Region Haná for Biotechnological and Agricultural Research, Institute of Experimental Botany, Sokolovska 6, CZ-77200 Olomouc, Czech Republic; 3 Centre National des Ressources Génomiques Végétales, INRA UPR 1258, 24 Chemin de Borde Rouge - Auzeville 31326 Castalnet Tolosan, France; 4 University of Haifa, Institute of Evolution and Department of Evolutionary and Environmental Biology, Haifa 31905, Israel; 5 Instituto di Genomica Applicata, Via J. Linussio 51, Udine, 33100, Italy; 6 MIPS/IBIS; Helmholtz-Zentrum München, 85764 Neuherberg, Germany

**Keywords:** chromosome 1BL, evolution, gene space, grasses, hexaploid wheat, map-based cloning, physical mapping, sequencing, synteny

## Abstract

**Background:**

As for other major crops, achieving a complete wheat genome sequence is essential for the application of genomics to breeding new and improved varieties. To overcome the complexities of the large, highly repetitive and hexaploid wheat genome, the International Wheat Genome Sequencing Consortium established a chromosome-based strategy that was validated by the construction of the physical map of chromosome 3B. Here, we present improved strategies for the construction of highly integrated and ordered wheat physical maps, using chromosome 1BL as a template, and illustrate their potential for evolutionary studies and map-based cloning.

**Results:**

Using a combination of novel high throughput marker assays and an assembly program, we developed a high quality physical map representing 93% of wheat chromosome 1BL, anchored and ordered with 5,489 markers including 1,161 genes. Analysis of the gene space organization and evolution revealed that gene distribution and conservation along the chromosome results from the superimposition of the ancestral grass and recent wheat evolutionary patterns, leading to a peak of synteny in the central part of the chromosome arm and an increased density of non-collinear genes towards the telomere. With a density of about 11 markers per Mb, the 1BL physical map provides 916 markers, including 193 genes, for fine mapping the 40 QTLs mapped on this chromosome.

**Conclusions:**

Here, we demonstrate that high marker density physical maps can be developed in complex genomes such as wheat to accelerate map-based cloning, gain new insights into genome evolution, and provide a foundation for reference sequencing.

## Background

Cereals crops, such as rice, maize, sorghum and wheat, are major caloric sources for humans and farm animals. While reference genome sequencesare available and are already supporting crop improvement in a challenging environment [1]for rice [2], sorghum[3]and maize [4], wheat genomics and its applicationis lagging behind. The wheat genome has always been viewed as impossible to sequence because of the large amount of repetitive sequences (>80%) [5],gigantic size (17 gigabases (Gb)) and the level of ploidy of bread wheat (2*n *= 6x = 42). Even with the rapid developments in DNA sequencing technologies that enable the production of gigabases of sequence within a few days [6], the short read lengths offered by these techniques and the large amount of repeated sequences present in the wheat genome make *de novo *assembly of non-genic regions extremely difficult [7]. These difficulties can be circumvented by focusing only on the gene catalogue and ignoring the intergenic regions that mostly consist of transposable elements. However, this practice is not justified in light of the results of whole genome functional analyses such as the characterization of 1% of the human genome in the ENCODE project [8] and association studies performed in maize [9]that clearly indicate the importance of intergenic regions in the regulation of genome expression. Thus, a complete wheat genome sequence is needed to access the complete catalogue of genes and regulatory elements and to provide a framework for understanding the impact of genomic variation on phenotypes. While long read single molecule sequencing may in the future enable tacklingof large and complex genomes using only whole genome shotgun (WGS) sequencing, the only feasible approach at this time to obtain a complete reference genome sequence of bread wheat is bacterial artificial chromosome (BAC) by BAC sequencing based on the construction of robust physical maps.

To reduce the complexity of physically mapping a 17 Gb hexaploid genome containing more than 80% similar or identical sequences, the International Wheat Genome Sequencing Consortium (IWGSC) [10] has adopted a strategy based on the individual sorting and analysis of chromosome or chromosome arms by flow cytometry [11] to construct specific BAC libraries[12]. The first BAC library [13]was used successfully to establish a chromosome landing-ready physical map of chromosome 3B, the largest wheat chromosome (1 Gb) [14]. This physical map has been used in several studies to analyze the composition and organization of the wheat gene space, provide estimates of the gene number, and determine the relative proportion of transposable elements families in the wheat genome[5,15,16]. In contrast to early cytogenetic studies based on expressed sequence tag(EST) mapping suggesting that most of the genes are found in a few large, gene-rich regions[17],these analysesrevealed the presence of numerous small gene islands dispersed along the chromosome and no geneless region larger than 800 kilobases (kb). Moreover, access to physical maps and sequences helped to refine collinearityrelationships between wheat and the other grass genomesby providing a higher level of resolution than genetic or cytogenetic mapping[15,16,18].The strategy used to build the physical map of wheat chromosome 3B was based on a high-information-content fingerprinting method [19] and FingerPrinted Contigs (FPC) software[20,21] for the assemblies. It resulted in 1,036 contigs with an N50 of 778 kb covering 82% of the chromosome[14]. To improve physical assembly in complex genomes, new software, called Linear Topological Contig (LTC),has been developed recently as an attractive alternative to FPC. It enables longer, better ordered and more robust contigs to be built compared to FPC contigs [22]. Physical maps are only useful when they are anchored to genetic maps and traits with markers. PCR methods used to anchor the physical map of chromosome 3B resulted in a marker density of 1.4 markers per megabase (Mb)and 56% of the physical map anchored. While useful for many map-based cloning projects, this marker density is far from that obtained in rice [23] or maize [24] (8 and 12 markers per Mb, respectively) and should be increased for breeding purposes. High throughput anchoring platforms [16]that increasethe number of genes anchored to the physical maps have been developed in wheat recently but more anchoring resources and efforts are still needed. In addition to anchoring the physical map with markers, it is important to order the physical contigs along the chromosomes. Here, the wheat genome is again a challenge because of the uneven distribution and lack of recombination in more than half of the chromosomes[25].

In this work, we used a combination ofnew high throughput genotyping assays and synteny with other grass genomes to establish a physical map of the wheat chromosome 1BL with the highest marker density for a wheat physical map so far (11 markers per Mb), a high level of anchoring (74% in the deletion bins; 19% on the genetic map) and a good percentage (48%) of contigs ordered along the chromosome arm. This physical map allowed us to gain new insights into chromosome evolution and refine estimates of physical sizes of deletion bins.Further, it provides a powerful tool for chromosome landing and for sequencing chromosome 1BL in the near future. The new high throughput marker assays combined with the optimized assembly and ordering methodologies proposed here can be applied to other plant genomes with similar levels of redundancy and complexity.

## Results

### FingerPrinted Contigs and Linear Topological Contig assemblies of the 1BL physical map

A 1BL-specific BAC library, containing 92,160 clones originating from sorted wheat chromosome 1BL of Chinese Spring and representing 15.4x coverage of the arm [12], was fingerprinted with the SNaPshot technology.A total of65,413 high quality fingerprints (71%) wasobtained and used to build a physical map. A first automated assembly was performed with the FPC software [20,21]following the guidelines adopted by the IWGSC[26]. This resulted in an assembly of 43,523 fingerprints into 3,030 contigs representing 807 Mb(151% of chromosome 1BL) with a N50 of 434 kb and a L50 of 391.A minimal tiling path (MTP) of 8,597 clones was designed and re-arrayed for further marker screening and analyses. Sixty-three -dimensional (plate, row and column) pools from the MTP and 240 plate pools from the whole 1BL BAC library were produced.During the course of the project, a new software -LTC [22] -specifically developed to build physical maps in complex genomessuch as wheat,became available.To improve the assembly of the 1BL physical map for future sequencing, we performed an automated LTC assembly using the same 65,413 high quality fingerprints. It resulted in an assembly of 41,940 fingerprints(including 94.4% in common with the FPC assembly) into 694 contigs representing 502 Mb (94% of the chromosome arm) with a N50 value of 961kb and a L50 of162. The maximum contig size was of 5,800 kb in the LTC map, three times longer than the1,780 kb in the FPC. This improved LTCmap was used as a template for adding the marker and order information and for building a final version of the map.

### A combination of high throughput approachesenables the construction of a 1BL physical map anchored with more 5,000 molecular markers

At the beginning of the project, there were only 171 1BL-specific PCR markers (114 single sequence repeats (SSR) and 57 restriction fragment length polymorphisms (RFLP)) available publicly in the GrainGenes database[27]. Thus, to develop a high density integrated physical map of chromosome 1BL, that is, a map comprising BAC contigs anchored to genetic and cytogenetic maps with a high number (>1,000) of molecular markers, we developed new molecular markers and anchored them to the 1BL physical contigs and genetic or cytogenetic maps.

The new 1BL markers were obtained from three different sources. The first source was2.17 million sequence reads obtained by the Roche 454 technology on amplified DNA from sorted chromosome 1BL of Chinese Spring[28], used to develop insertion site-based polymorphism (ISBP) [29]and SSR markers. In total, 46,606 high confidence and non-redundant markersincluding 46,194 ISBPand 412 SSR markers were designed automatically by the IsbpFinder.pl[29] and ssrFinder.pl programs, respectively. From these, 1,200ISBPs and 200 SSRswere randomly selected for direct PCR screening (see below) of the three-dimensional pools of the 1BL MTP. To increase the throughput, we also developed a new platform for ISBP markers by designing an ISBP-1BL-specific NimbleGen microarray. To do this, sequences corresponding to junctions between a transposable element (TE) and a low-copy sequence were specifically selected among the 46,194 ISBP markers designed above. In total, 17,788 of such ISBP markers, including 193 in common with the 1,200 PCR-based ISBPs,were used to design a 17k 1BL ISBP array that was then hybridized with the MTP pools (see below). Thus, 18,795 ISBP and 200 SSR markers designed from 454 sequence reads of sorted 1BL chromosome were used for screening the three-dimensional MTP pools.The second source of markers originated from hybridization of the three-dimensional MTP pools with the wheat NimbleGen 40k unigene microarray that was developed previously by Rustenholz *et al.*[15].Finally, 445 conserved orthologous set (COS) markers identified on chromosome group 1L (1AL, 1BL and 1DL) from an ancestral set of 10,000 grass COS markers defined by comparative studies between wheat, barley, rice, sorghum and *Brachypodium*[30] were used as a third source of markers for direct PCR screening of the MTP pools. Such COS markers should enable the 1BL map to be linked directly to other grass genomes through the ancestral gene set.

**Figure 1:**
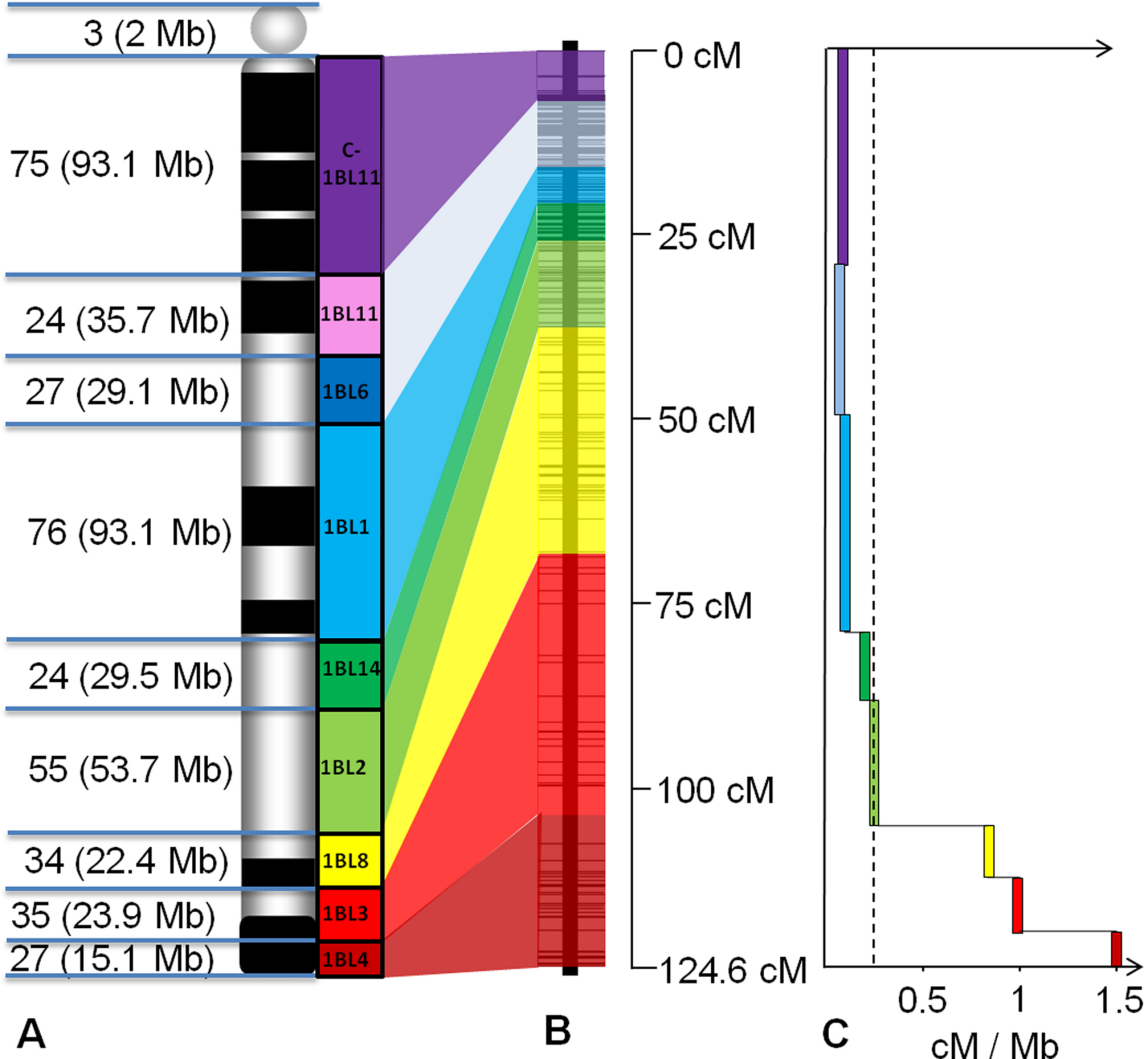
**Representation of the integrated physical and genetic map and distribution of recombination rate along wheat chromosome 1BL**. **(A) **Representation of the 1BL deletion bin map. The centromere is represented as a grey circle and the nine deletion bins are represented by colored boxes as follows: C-1BL11-0.23 deletion bin in purple, 1BL11-0.23-0.32 in pink, the 1BL6-0.32-0.47 in blue, 1BL1-0.47-0.61 in light blue, 1BL14-0.61-0.69 in green, 1BL2-0.69-0.74 in light green, 1BL8-0.74-0.85 in yellow, 1BL3-0.85-0.89 in red and1BL4-0.89-1.00 in dark red. The number of physical contigs assigned to a bin and the cumulative size of these contigs are indicated. When contigs carried BACs that were assigned to two different consecutive bins indicating that they likely are at the junction between the bins, the contig was counted for 0.5 in each bin. **(B) **Representation of the 1BL neighbor genetic map. The map is divided into segments corresponding to the deletion bins except for deletion bins 1BL11-0.23-0.32 and 1BL6-0.32-0.47 that were merged. **(C)**Representation of the ratio between the genetic and the physical distances along the 1BL chromosome using physical contigs to estimate the bin sizes. The dotted line corresponds to the average ratio on the whole chromosome arm. Values are expressed in cM/Mb.

All molecular markers were then used for cytogenetic and genetic mapping on chromosome 1BL. A total of 1,611 PCR markers, including the newly developed 1,200 ISBP and 200 SSR, the 171 publicly available PCR markers, and 40 of the 445 COS corresponding to genes not present on the wheat NimbleGen 40k unigene microarray, werefirst checked for their specificity to wheat chromosome1BL using aneuploid lines from cv Chinese Spring,including a nullisomic-1B-tetra-somic 1A line, the 1BL and 1BS ditelosomic lines[31,32]. Out of the 1,611 tested markers,594(37%) were clearly 1BL-specific. To obtain further indication of their position along the chromosome,the 1BL-specific markers weretested on genomic DNA of eight deletion lines representing nine deletion bins. In total,549 markers (92%;475 ISBP, 67 SSR, 4 RFLP and 3 COS) were unambiguously assigned to one of the ninebins (Table 1). In addition, 84 markers (36 ISBPs and 48 SSRs) showing polymorphism between Chinese Spring and Renan, the parents of a 381 F2 mapping population (CSReF2), were mapped genetically. Linkage analysis resulted in a genetic map of 124.6 centimorgan (cM)with an average of one marker every 1.5 cM. Ahigh markerdensity was observed in the proximal part of the chromosome, with 33 markers (39%) located in the first 10 cM of the map. Following the method used for chromosome 3B[14], we built a 1BL neighbor genetic map using the Chinese Spring ×Renanmap as a basis and sevenadditional genetic maps from fivebi-parental populations andtwoconsensus maps representing 13 populations (see Materials and methods).The 1BL neighbor map consisted of 478 markers including 223 SSRs, 97 genes, 80 RFLPs, 50 ISBPs, 26 Diversity ArraysTechnology, 1 single nucleotide polymorphism and 1 protein marker(Figure 1B). This represents a density of onemarker per 0.26 cM.

**Table 1:** Distribution of the number of markers, genes and physical contigs in 9 deletion bins along the centromere-telomere axis of chromosome 1BL

	Markers assigned^a^	Contigs^b^	Contigs size^c^	Markers^d^	Genes^e^	Gene density^f^	Non-syntenic genes^g^	Genes in islands^h^
Centromere	3	02.5	02.03	6	2	0.98	2	1
C-1BL11-0.23	115	75.5	93.15	722	168	1.80	99	115
1BL11-0.23-0.32	37	24.0	35.67	354	77	2.16	52	48
1BL6-0.32-0.47	36	27.0	29.05	247	68	2.34	24	37
1BL1-0.47-0.61	109	76.0	93.14	1160	232	2.49	114	158
1BL14-0.61-0.69	34	24.0	29.52	311	80	2.71	57	65
1BL2-0.69-0.74	79	55.0	53.71	714	155	2.89	102	105
1BL8-0.74-0.85	48	34.0	22.35	418	66	2.95	52	48
1BL3-0.85-0.89	51	35.5	23.88	507	81	3.39	47	62
1BL4-0.89-1.00	37	26.5	15.10	302	57	3.77	40	45
Total	549	380	397.6	4741	986	2.48	587	683

^a ^Number of markers assigned to the deletion bins; ^b^number of contigs assigned to the deletion bins; ^c^cumulated size of the assigned contigs in Mb; ^d^total number of markers in the assigned contigs; ^e^number of genes in the assigned contigs; ^f^the gene density in genes per Mb was estimated in each bin using the number of genes anchored divided by the cumulated contigs size; ^g^genes not conserved in syntenic regions of rice, *Brachypodium *or sorghum are indicated for each bin; ^h^the number of gene islands was calculated for each bin using the number of genes in the same BAC or overlapping BACs in each bin.

To establish links between the physical, geneticand cytogenetic maps, we first screened the 63 three-dimensional pools of the MTP and the 240 plate pools of the complete 1BL BAC library with 465 1BL-specific PCR. Of these, 431 markers amplified at least one MTP row, column and MTP plate pool and/or several plate pools. Deconvolution of the information was performed using a homemade Perl script (named DSMP.pl) for the MTP pools results and the Elephant (*ele*ctronic *ph*ysical map *an*choring *t*ool) [33]software for the plate pools results (see Materials and methods). This led to the assignation of 416PCR markers (320 ISBPs, 70 SSRs, 22 COSs and 4 RFLPs) to individual BACs (Additional file 1). The information was integrated into the final version of the physical map obtained by LTC, resulting in the anchoring of 241 contigs with the 416 markers.

The 40k unigene and 17k 1BL-ISBP NimbleGen arrays were hybridized with the 63 three-dimensional MTP pools. After signal quantification, normalization (see Materials and methods) and data deconvolution, 3,912 ISBPs and 1,615 unigenes were unambiguously assigned to individual BACs and subsequently to contigs of the LTC map. Ten ISBPs were randomly selected to control the accuracy of the assignation using microarray hybridizations.In nine cases, the putative positive poolswere confirmed,demonstrating the robustness of the methodology.To confirm the identity of the putative 1BL genes identified by hybridization of the 40K unigene array, we performed two additional controls: hybridization of genomic DNA from sorted chromosome 1BL on the array, and identification of the 1BL unigenes of the array by sequence similarity with the Roche454 sequence reads obtained from sorted chromosome 1BL.Using these data, 392 unigenes assigned to the physical contigs by hybridization of the three-dimensional MTP pools but not confirmed by the 454 reads or by hybridization of the sorted chromosome 1BL genomic DNA were discarded to avoid any ambiguity.This resulted in 1,223 unigenes assigned with high confidence to the 1BL physical contigs.

To eliminate redundancy in the unigene set, weused information from orthologous genes in rice and *Brachypodium distachyon *as well as the latest release of the wheat unigene build (version 59).This resulted in the elimination of 62 redundant unigenes from the 1BL physical map, leading to a total of 1,161 unique genes unambiguously assigned to the 1BL physical map. Thus, together with the 4,232 ISBPs, 70 SSR, 22 COS and4 RFLP, the1,161 unigenes provide a 1BL physical map anchored with 5,489 markers (Additional file 1).

The marker information was then used to guide a manual assembly step for the physical map (see Materials and methods). This enabled themerging of 78 contigs,thereby resulting in a final 1BL physical map of 616 contigs representing 497 Mb (93% of the chromosome arm)with a N50 of 1128 kb, a L50 of 142 and a density of markers of 11 markers per Mb. To order the physical contigs of the 1BL map along the chromosome, we identified the contigs carrying the 543 markers located in the nine deletion bins as well as the 84 markers from the Renan × Chinese Spring genetic map. This enabled the placement of 380 of the 616 contigs representing 74% of chromosome 1BL (397.6 Mb),including 986 genes (84.9% of the 1,161 wheat 1BL unigenes),in thenine deletion bins, and 84 contigs representing 19% of chromosome 1BL (103 Mb) on the 1BL neighbor genetic map (Figure 1A; Additional file 2).Three contigs representing 2 Mb were assigned specifically to the centromere (Figure 1A).The anchored physical map of wheat chromosome 1BL is available from the Unité de Recherche Génomique Info website [34].

### Physical to genetic map ratio analyses reveal discrepancies in the estimation of the deletion bin sizes between the physical and cytogenetic maps

**Figure 2:**
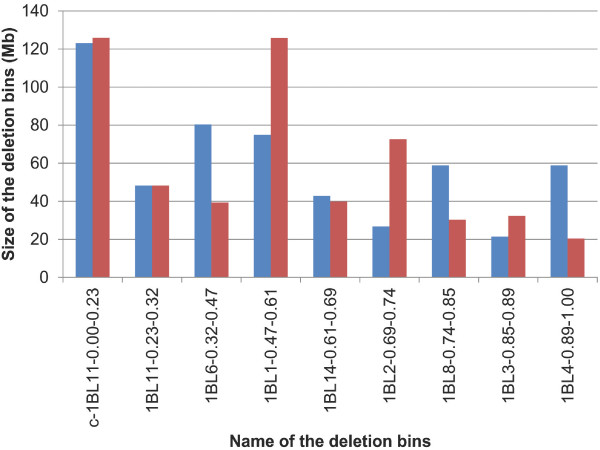
**Comparison of the size ofnine deletion bins along chromosome 1BL using cytogenetic and physical estimations**. The cytogenetic and physical estimates are provided in blue and red, respectively. The size of each bin is provided on the Y axis in Mb. Bins are ordered from the left to the right along the centromere-telomere axis.

The anchored physical map was used to measure the ratio between physical and genetic distances and study the recombination pattern along chromosome 1BL. We first estimated the size of the nine deletion binsusing the cumulative size of the physical contigs anchored in each bin corrected by the estimated percentage of chromosome coverage (74%). It ranged from 20 to 125 Mb(Figure 2). When compared to the estimations based on cytogenetic measurements[31,32,35],some striking differences were observed (Figure 2). While three deletion bins (C-1BL11-0.23, 1BL11-0.23-0.32 and 1BL14-0.61-0.69) had very similar values, the sizes of the contiguous 1BL6-0.32-0.47 and 1BL1-0.47-0.61 deletion bins were respectively overestimated (49 %) and underestimated (60 %) by the cytogenetic estimation. Interestingly, the cumulated sizes of the two bins were similar in the two estimates (only 6% of difference), suggesting an error in the measure of the limit between the two deletion bins by the cytogenetic analysis. A similar difference was observed with the contiguous 1BL2-0.69-0.74 and 1BL8-0.74-0.85 deletion bins (16% difference between the two estimations of the cumulative size of the two deletion bins). The largest discrepancy (34%) was observed for the most distal bin 1BL4-0.89-1.00.

Based on an overall estimated size of 535 Mb for chromosome 1BL [12] and a genetic map of 124.6 cM, the average genetic to physical distance ratio is 0.23 cM/Mb. The ratio between the genetic and physical distances was calculated further for each deletion bin using size estimates obtained from the physical maps. For this analysis, the 1BL11-0.23-0.32 and 1BL6-0.32-0.47 deletion bins were merged because it was impossible to identify unambiguously the limit between them on the 1BL genetic map (Figure 1B). The pattern of the cM/Mb ratio along chromosome 1BL revealed three main parts. The first one, representing 63% of the chromosome (from the centromere to the 1BL1-0.47-0.61), had a ratio close to 0.05 cM/Mb on average (Figure 1C). In the second part, representing 21% of the chromosome (1BL14-0.61-0.69 and 1BL2-0.69-0.74 deletion bins), the ratio increased to 0.20 cM/Mb on average (Figure 1C); whereasin the remaining 16% of chromosome 1BL,corresponding to the telomeric part, the average ratio dramatically increased toabout 1 cM/Mb and up to 1.46 cM/Mb in the most telomeric 1BL4-0.89-1.00 deletion bin.

### Synteny-based approaches to establish a putative gene order along wheat chromosome 1BL

**Figure 3:**
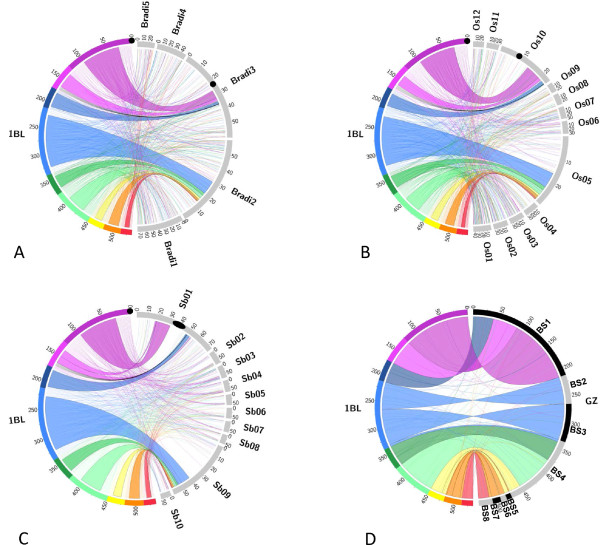
**Schematic representation of the syntenic relationships between wheat chromosome 1BL and the orthologous chromosomes in *Brachypodium distachyon*, rice and sorghum as well as the 1BL GenomeZipper**. **(A) ***B. distachyon*, **(B) **rice and **(C) **sorghum chromosomes in which syntenic regions were identified are represented in grey on the right side of the circle. Large areas represent the syntenic regions identified with each species while individual colored lines identify the non-syntenic genes. The black lines represent a wheat specific genome rearrangement. Black circles correspond to the centromeres of wheat chromosome 1BL, Bradi3, Os10 and Sb01. **(D) **Comparison between the 1BL virtual gene order based on the 1BL physical map (left part of the circle) and on the GenomeZipper approach (right part of the circle). Each line provides a link between the positions of the same gene on the two virtual gene ordering. The nine deletion bins of wheat chromosome 1BL are represented in colors on the left side of the circle:C-1BL11-0.23 deletion bin in purple, the 1BL11-0.23-0.32 in pink, the 1BL6-0.32-0.47 in dark blue, the 1BL1-0.47-0.61 in blue, the 1BL14-0.61-0.69 in dark green, the 1BL2-0.69-0.74 in green, the 1BL8-0.74-0.85 in yellow, the 1BL3-0.85-0.89 in orange and the 1BL4-0.89-1.00 in red.

Using the information from marker anchoring in the nine deletion bins, we were able to propose a rough order along chromosome 1BLfor 380 contigs including 986 genes.However, within each bin, it was impossible to assess the relative order of the contigs except for those 84 that were anchored on the genetic map. To progress further in ordering the contigs and to analyze the gene space distribution, we used the synteny between the genes located on the 1BL physical contigs and the genome sequences of rice, *B. distachyon*, and sorghum.Among the 986 genes assigned to the 1BL deletion bins, 815 homologous genes were identified by sequence alignments (see Materials and methods) in *B. distachyon*, 816 in rice and 834 in sorghum (Figure 3A-C; Additional file 1).The 815 homologous *Brachypodium *genes were found on the five *Brachypodium *chromosomes with a majority (74.5%) on chromosome Bradi2 (354 genes) and Bradi3 (253 genes) (Figure 3A). Out of the 354 genes on Bradi2 and 236 genes on Bradi3, respectively 236 (66.7%) and 128 (50.6%) formed unique collinear blocks defining syntenic regions on these chromosomes (Figure 3A). In rice, 206 and 103 of the 816 homologous genes were found in single collinear blocks on rice chromosomes 5 and 10, respectively (Figure 3B). In sorghum, 329 of the 834 homologous genes were found in three collinear blocks including two on chromosome Sb01 (65 and 44 genes) and one on chromosome Sb09 (220 genes) (Figure 3C). Interestingly, the disruption of collinearity observed on chromosome Sb01 corresponds to the centromeric region. When compared to the position and size of the centromeric regions of chromosome 1BL, Bradi3 and Os10 (Figure 3), the results suggest a shift of at least 13 Mb of the Sb01 centromere and an expansion of the centromeric regionthat is likely due to the accumulation of LTR-retrotransposons, as observed previously for all sorghum chromosomes[3]. Thus, these results show a clear breakpoint in the synteny between the genes located in the first three deletion bins of chromosome 1BL and those in the remaining six deletion bins. The first region corresponds to chromosome Bradi3, Os10 and Sb01,andthe second corresponds to chromosomes Bradi2, Os5 and Sb9.

In total, 399 of the 1,161 (40.5%) 1BL genes corresponding to 181 contigs were syntenic with rice, sorghum or *B. distachyon*;whereas, 587 (59.5%) genes corresponding to 234 contigs were non-syntenic (Table 1; Additional file 1). Among the non-syntenic genes, there was no significant bias towards any of the other chromosomes in the three grass species (Figure 3A-C;Additional file 1).

A putative order of physical contigs within the deletion bins was established for the contigs carrying syntenic genes based on the order of their ortholog in at least one of the three grass species (Figure 3D).This provided an order for 180 contigs representing 48% of chromosome 1BL (257 Mb) (Additional file 2).For 10 contigs (15 syntenic genes), the deletion bin information wasinconsistent with the synteny,indicating putative rearrangements.Seven of the contigs were anchored with a single gene and thereforethere was insufficient information to conclude potential rearrangements between wheat and the other genomes. The three remaining contigs (4.6 Mb of total size)were anchored in deletion bin 1BL11-0.23-0.32 and corresponded to single blocks in rice (Os10g0573800 to Os10g0576000), *B. distachyon *(Bradi3g34090 to Bradi3g34400) and sorghum (Sb01g28100 to Sb01g28320) that are syntenic with deletion bin 1BL6-0.32-0.47(Figure 3A-C). This result indicates an intra-chromosomal translocation of a regionof at least 4.6 Mb in the wheat lineage.

In a second step,we established a putative order along the wheat chromosome 1BL for the genes located in the 180 ordered contigs usingthe relative position of the BACs within the contigs. This resulted in ordering 787 genes (68% of the 1BL genes set) along the 1BL physical map (Additional file 1).To check this putative gene order, we compared it to a gene order obtained by the GenomeZipper[36,37] approach, in which a virtualgene orderis built using synteny information and genetic mapping. A zipper of the wheat chromosome 1BL was performed by integrating information from a wheat 1BL genetic map comprising 242 gene-based single nucleotide polymorphism markers obtained by genotyping by sequencingof the International Triticeae Mapping Initiative (ITMI) population[38], 198,968 sequence contigs from the Illumina sequencing of sorted wheat chromosome 1BL performed by the IWGSC [10], wheat ESTs from the HarvEST database [39], barley full-length cDNA, and the rice, sorghum and/or *B. distachyon *genes (Additional file 3).The 1BL zipper provided a virtual order for1,593wheat loci including 1,433 genes organized in eightsyntenic blocks containing between 29 and 506 genes (average = 179) and 2.6 markers per block (min = 1, max = 8) (Figure 3D;Additional file 3).In total, 429 genes with an average of 51 genes (8 to 132) per syntenic block were shared between the physical map-based and zipper-based virtual gene orders. Of these genes, 354 (82.5%) were found in the exact same order in the two maps. However, the relative orientation of blocks BS1, 2, 3, 5 and 7in the zipper was completely invertedcompared to the bin order(Figure 3D). A closer look at the genetic mapping data of the 1BL zipper revealed that in these syntenic blocks, the numberof markers and recombination events was very low (Additional file 3),thus leading to unreliable orientations of the blocks. In the case of BS1 and BS5, the information of the physical map clearly demonstrates that the genetic map is erroneous (Figure 3D). These results suggest that the resolution and accuracy of the gene-based wheat genetic maps remains a limiting factorfor reliable ordering of wheat physical maps using synteny with the other grass genomes.

With about half of the BAC contigs and 68% of the genes ordered, the physical map of chromosome 1BL provides a unique resource for detailed analyses of the gene space, accelerated map-based cloning,and future chromosome sequencing.

### Gene space organization and evolution of wheat chromosome 1BL

**Figure 4:**
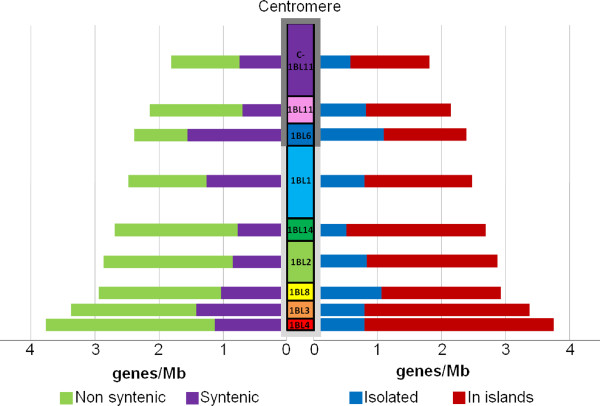
**Gene distribution along wheat chromosome 1BL**. The gene density in each of the nine deletion bins is indicated in gene/Mb on each side of the schematic representation of the chromosome. The left end side presents the relative percentage of syntenic and non-syntenic genes per bin whereas the percentage of genes found in islands versus isolated is indicated for each bin on the right end side. Bins are represented with the following color code:C-1BL11-0.23 deletion bin in purple, the 1BL11-0.23-0.32 in pink,1BL6-0.32-0.47 in blue, 1BL1-0.47-0.61 in light blue, 1BL14-0.61-0.69 in green, 1BL2-0.69-0.74 in light green, 1BL8-0.74-0.85 in yellow,1BL3-0.85-0.89 in orange and 1BL4-0.89-1.00 in red. In wheat, chromosomes of group 1 originate from the fusion between the ancestral proto-chromosomes A5 and A10 [40]. The parts of chromosome 1BL that originated from proto-chromosome A10 and A5 are marked by dark grey and light grey boxes, respectively.

The 986 genes assigned to one of the nine wheat chromosome 1BL deletion bins allowed us to calculate the gene density per deletion bin using the cumulated length of anchored contigs in each deletion bin (Table 1). The results show that the gene density distribution correlates with the distance from the centromere (Pearson's correlation coefficient r = 0.956, *P *= 5.10^-5^), demonstrating the presence of a gradient of gene density with a two-fold increase from the centromere (1.8 genes/Mb in C-1BL11-0.23) to the telomere (3.77 genes/Mb in 1BL4-0.89-1.00; Figure 4).We then examined the correlation between the distribution of gene density and the density of syntenic and non-syntenic genes per deletion bin (Figure 4). This revealed that the gradient of gene density along chromosome 1BL is mainly due to the presence of non-syntenic genes (Pearson's correlation coefficient r = 0.870, *P *= 0.0022) whereas the distribution of syntenic genes has no impact on the overall gradient (Pearson's correlation coefficient r = 0.370, *P *= 0.326; Figure 4). Thus, these results indicate a gradient of gene density from the centromere to the telomere of chromosome 1BL that is correlated with the proportion of non-syntenic genes.

To further investigate the gene space organization, we studied the proportion and distribution of gene islands along the chromosome arm. Gene islands were defined as regions in which genes are located on the same or overlapping BACs. A total of 683 genes (69%) were identified as genes in islands and formed 284 gene islands composed of two to eight genes (average = 2.9 ±1.2, median = 2). Such a proportion of genes in islands is higher than the percentage expected from a random distribution as revealed by 10,000 random samplings without replacement of 1,161 genes on chromosome 1BL BACs (average = 54% ±1.6%, *P *= 10^-15^by χ^2 ^test). The remaining 304 genes (31%) were considered as isolated genes whose density was shown to not be correlated with the distribution of total gene density (Pearson's correlation coefficient r = 0.182, *P *= 0.638). The proportion of genes in islands varied among deletion bins (ranging from 54% in 1BL6-0.32-0.47 to 81% in 1BL14-0.61-0.69) and the density of genes in islands wascorrelated positively with the distribution of the total gene density (Pearson's correlation coefficient r = 0.962, *P *= 0.000033; Figure 4). Moreover, this density wasalso correlated with the density of non-syntenic genes (Pearson's correlation coefficient r = 0.916, *P *= 0.00051) whereas no correlation wasfound with the density of syntenic genes (Pearson's correlation coefficient r = 0.208, *P *= 0.590). These results show that the gradient of gene density observed along chromosome 1BL results from an increase of genes in islands along the chromosome axis from centromere to telomere.

As mentioned above, the general pattern of the distribution of syntenic and non-syntenic genes along the chromosome showed an increase in the proportion of non-syntenic genes from the centromere to the telomere. However, an additional peak of synteny was observed in the proximal bin 1BL6-0.32-0-47 (65%; Figure 4).

In wheat, chromosomes of group 1 and group 3 originate from an ancestral duplication of one of the five proto-chromosomes (A5) identified by Salse *et al.*[40] during paleogenomics studies of the grass genomes. Here, we wanted to investigate how many of the 1,161 genes found on chromosome 1BL are still conserved on chromosome 3BLand originate from the ancestral proto-chromosome A5. Toidentify these genes,we usedthe ancestral grass gene set defined by Murat *et al.*[41], that is, genes conserved among all grass genomes. We identified128 rice genes corresponding to 64 genes duplicated between rice chromosomes 1 and 5 that also derive fromthe ancestral proto-chromosome A5[41]. Their coding regions were alignedbytblastx analysis against the Illumina contigs from the IWGSC survey sequencing of all wheat chromosome arms. All wheat genes matching an ancestral rice gene with 35% of identity at the amino acid level on 70% of the length of the rice genes were considered as putative homologs to the ancestral gene. Nineteen of the 128 rice genes matched more than 12 wheat chromosome arms andwere eliminated from the analysis as they were considered belonging to large multigene families. We found 18 homologs to ancestral duplicated gene pairs on rice chromosomes 1 and 5 located on wheat chromosome 1AL, 14 on 1BL and 10 on 1DL, corresponding to 21 non-redundant genes in chromosome group 1L (Table 2). In chromosome group 3L, 12 homologs were found on wheat chromosome 3AL and nineon 3DL. For chromosome 3B,the survey sequences correspondedto theentire chromosome (and not to the two arms separately), and 38 homologs were identified including 12that were homologous to the same rice chromosome 1 and chromosome 5 ancestral duplicated pairs as the 1BL genes. These 12 genes were considered to belocatedon the long arm of wheat chromosome 3B (Table 2).This was confirmed by ananalysis of the chromosome 3B reference sequence recently established by our laboratory (unpublished data).All together, 19 non-redundant genes were found on chromosome group 3L.Using these results, we identified ninegenes from the ancestral duplications between wheat chromosome 1AL and the 3AL, 12 between 1BL and 3BL, and sixbetween 1DL and 3DL, leading to 16 non-redundant genes from the ancestral duplication between group 1L and 3L. The 1,161 gene set anchored to the 1BL physical map contained eightof the 12 ancestrally duplicated genes identified between wheat chromosome 1BL and 3BL (data not shown), demonstrating that there are a few genes from the ancestral grass genome duplication that can still be detected.Interestingly, the 64 genes from the ancestral proto-chromosomeA5 that are still conserved between rice chromosomes 1 and 5 represent1.2% and 2.1%of the total gene content for the two chromosomes, respectively (5,078rice chromosome 1 and3,118 rice chromosome 5 non-TE-related genes).Bycontrast,in wheat, these conserved ancestral genes represent only0.32% of chromosome 1BL and 0.25% ofchromosome 3BL genes, based on estimations of3,700 genes [28]on 1BL and4,700 on 3BL[18], thereby suggesting a higher level of rearrangement during the evolution of the wheat genome than for rice.

**Table 2:** Number of wheat genes originating from the grass ancestral duplication identified on wheat chromosomes group 1 and 3

Wheat chromosome or chromosome arm	Number of wheat genes originating from the grass ancestral duplication^a^
1AL	18
1BL	14
1DL	10
Group 1L	21
3AL	12
3BL	12
3DL	9
Group 3L	19
Other long arms of wheat chromosomes	3.4 ±2.5
Other groups L	6.4 ±3.5

^a ^Chromosomal distribution of the 64 ancestral duplicated genes pairs from rice chromosomes 1 and 5 matching the Illumina contigs from the IWGSC survey sequencing of all wheat chromosomes.

### A physical map with more than 5,000 markers to support efficient map-based cloning on chromosome 1BL

The high quality of the 1BL physical map (616 contigs covering 93% of the chromosome arm), the high number of markers (5,489 including 1,161 genes) anchored to the physical map and the good percentage of ordered contigs (48% of the chromosome arm) provide a robust platform for supporting map-based cloning. Currently,40 quantitative trait loci (QTLs) involved in various agronomically important traitssuch as resistance to biotic stress, nitrogen use efficiency or bread-making quality have been mappedon wheat chromosome 1BL (as of July 2012) [42]. The average confidence interval of these QTLs is6.68 cM. Based on a ratio of 0.06 cM/Mb in 70% of the centromeric part of the 1BL genetic map and 0.6 cM/Mb in the remaining part, the average confidence interval of these QTLs is about 83 Mb. Thus, with the current marker density of the 1BL physical map, each QTL interval contains potentially 916 markers including 193 genes. The potential of the1BL map for positionalcloningcan be illustrated with a representative example from the literature. For a QTL found for bread volume (Bvol[43])that was mapped on chromosome 1BL in a confidence interval of 11.8 cM flanked by markers wmc156 and gwm403, our integrated physical map provides 50 ordered physical contigs, containing 1,066 markers including 248 genes, and 105 unordered contigs, containing 1,113 markers including 219 genes for further fine mapping and positional cloning. With such information tohand, it is possible to define a subset of markers ordered along the interval and initiate fine mapping in a high resolution recombinant populationto rapidly identify physical contigs that can provide additional marker and gene candidates or be sequenced. When the QTL is refined to smaller intervals and locatedin regions with a ratio of 0.6 cM/Mb, as is the case for nine QTLs currently mapped within an estimated confidence interval of less than 3 cM on chromosome 1BL, the potential of the 1BL physical map becomes even higher. Indeed, with an N50 value for the contig size of 1.1 Mb, oneto three contigs should be sufficient to cover such intervals, thereby providing landing pads for gene isolation.

## Discussion

### Challenges and new opportunities for constructing and anchoring physical maps in wheat

Because ofits size (17 Gb) and complexity (polyploid (2n = 6x =42) with a large amount of repetitive sequences (>80%))[18], the wheat genome has been viewed as 'impossible' to physically map and sequence.Recently, the construction of a physical map of the largest wheat chromosome (3B) demonstrated the feasibility of physical mapping in hexaploid wheat using a chromosome-based approach[14].Here, using the same tools (SNaPshot, FPC)andassembly methodology, we built a first automated assemblyof the 1BL physical map. It resulted in a coverage of 151% of the chromosome arm,which suggeststhat a lot of small contigs covered the same regions as larger contigs.Such a result can be explained by fingerprints of low quality. In fact, atechnical problem occurred on the automated sequencer during fingerprinting leading to low reproducibility.As initial assemblies with FPC are performed at high stringency (cut-off of 1e^-75^) andBAC fingerprints are merged into contigs only if they share more than 70% of the bands [14], low quality fingerprints result in BAC clones from the same regions that are not merged, thereby increasing the number of contigs for a given region. Fingerprinting in wheat is not trivial. A number of critical steps (bacterial growth conditions, restriction enzymes quality, running conditions) for producing high quality fingerprints have been identified by key laboratories involved in wheat fingerprinting [26]. One of the important factors is to perform the fingerprints in a comparable manner throughout the duration of the experiment (that is, several months)and using the same sequencer is strongly recommended.Decreasing the initial stringency of the FPC assembly to compensate for lower fingerprint quality is not an optionin wheat because the high percentage of repetitive sequences leads to a large numberof chimerical contigs [14]. Dealing with such problems and improving assembly in wheat was one of the rationales behind the development of theLTC software [22].Here, we demonstrated with a real case study that LTC improves assembly even with a suboptimal quality of fingerprints. Assembly with LTC resulted in a robust 1BL physical map covering 94% of the chromosome arm and with an N50 of 1,128 kb, that is,about threetimes longer than with FPC andgenerally higher than the values achieved so far with FPC in wheat and barley[14,44].

Whole Genome Profiling (WGP™) that relies on sequence-based fingerprinting of BACs to buildphysical mapshas been developed recently [45] and was evaluated for wheat using a subset of the wheat 3B chromosome BAC library [46].The results showed that, with an improved assembly methodology, the WGP-based physical map contained 30% fewer contigs than the SNaPshot physical map with an equivalent coverage of the target regions, and that the WGP contigs contained 3.5 times less mis-assembled BACs. Moreover, further improvements of the methods by using *Eco*RI instead of *Hin*dIII for the restriction digest and increasing the length of the sequence tags using longer reads were proposed [46]. Thus, based on the experience with the physical map of chromosome 1BL and the WGP pilot study on chromosome 3B,we recommend combining the improved WGP fingerprinting methodology with the LTC assembly software for future physical mapping efforts in wheat.

To achieve the full benefit of a physical map, BAC contigs need to be anchored and ordered to genetic and/or cytogenetic maps with molecular markers. PCR-based methods coupled with pooling strategies arecost effective to perform such anchoring. Here, we used the same pooling scheme (three-dimensional pools) as forthe 3B physical map [14], but with an improved method using a new Perl script called DSMP.pl to automate the deconvolution of the three-dimensional MTP pool screening results. This enabled us to deconvolute the information for89.5% of markers.Moreover, the hybridization of the three-dimensional 1BL MTP pools on gene-based (40k) and ISBP-based (17k) arrays allowed us to place more than 5,000 markers on the physical map of chromosome 1BL. This represents a marker density of 11 markers per Mb, the highest density of markers obtained to date for a wheat physical map. The marker density on the 1BL physical map is about 10 times higher than the first version of the 3B physical map (1.4 marker/Mb) [14] and the number of contigs anchored and ordered represents 74% of chromosome 1BL, compared with only 56% of chromosome 3B. This marker density is equivalent to the density obtained for the maize physical map (12 markers per Mb) [24] and is even higher than the density obtained forthe rice physical map (8 markers per Mb) [23] and the recent barley physical map (0.65 markers per Mb) if we exclude the markers placed *in silico*[44].

One of the recurrent difficulties in wheat physical mapping is the lack of precision in orderingmarkers along the chromosomes. Until recently, ordering in wheat was performed using deletion bins ranging from 20 to 125 Mbin size [47,48], thereby making it nearly impossible to assess the order of contigs or markers within bins. In addition, estimates of the deletion bin sizes thathave been used to calculate physical to genetic distances ratio (cM/Mb) in wheat are prone to errors as demonstrated in this study.Ourresults suggest inaccurate estimates of the bin sizes by cytogenetic measurements, with increased errors at the telomeric ends of the chromosome thatarelikely due to higher decondensation of the DNA in these regions. Cytogenetic measurements of the deletion bin sizes were initially performed on photographs with rulers, which can largely explain the inaccuracy [35]. Alternatively, it is possible that the size of some deletion bins estimated by the physical map is underestimated due to under-representation of the corresponding region in the BAC library. Thus, these results confirm that, in wheat, the recombination rate is very low in 70% to 80% of the chromosome and is multiplied by 10 to 20 in the remaining 20% to 30% with a steady increase towards the telomeres. They suggest also that the construction of physical maps provides a reliable substitute to deletion bins for performing accurate recombination studies, more particularly in the telomeric regions where the cytogenetic estimation seems erroneous.

Ordering physical contigs can be supported to some extent by genetic mapping. Here, we obtained a genetic map of 124.6 cM, which is in the range of the length reported for 1BL in the literature [48-50]. However, recombination is not evenly distributed along the wheat chromosomes and in about two-thirds of the chromosome length, recombination is severely reduced or absent [25]. This makes it impossible to order about two-thirds of the wheat physical maps, especially when using bi-parental genetic maps with small size populations [14,25,48,51,52]. To overcome this problem, we developed a strategy that combinesgenetic mapping with deletion bin assignation and synteny with rice, *B. distachyon *and/or sorghum. The use of synteny in grasses to order BAC contigs and genes is based on the fairly high collinearity observed between the cereal genomes [53,54]. Here, we confirmed this high collinearity by the good correlation between the deletion bin assignation of the 1BL unigenes and the order of the orthologous genes in rice, sorghum and *B. distachyon*. One exception was observed for three contigs that likely reflects an intra-chromosomal translocation of a region of at least 4.6 Mb in the Triticeae lineage. The main limit of the synteny approach concerns the conservation at the micro-collinearity level. Several studies demonstrated a very good conservation of the micro-collinearity between cereals [55,56] but others showed some local rearrangements [57-59]. Here, 48 ordered BAC contigs contained more than three syntenic genes, for a total of 195 syntenic genes. Only 12 out of these 195 syntenic genes (6%) were not in the same order in wheat compared to the other grasses (data not shown). Because some of these differences may be due to errors in the ordering of BACs in the contigs by FPC, we estimate a maximum of 6% break in the micro-collinearity between wheat chromosome 1BL and the orthologous regions in the others grass genomes.

The comparison of our anchoring strategy with the GenomeZipper strategy[36,37], which integratesgene-based genetic mapping information usingsynteny toother grass genomes, demonstrated thatthe difficulty in mapping homoeologous genes unambiguously and the lack of recombination remain aserious limitation for ordering physical maps in hexaploid wheat. Two others strategies can be deployed to overcome the lack of recombination in wheat bi-parental genetic maps. The first one consists ofsimultaneously increasing the number of meiosis and, thus, the number of recombination and polymorphism by using more than two parents.Multi-parent advanced generation inter-cross (MAGIC) populations can be developed by intercrossing a number of parent lines (2^n^) for n generations in a set mating designed to combine the genomes of all (2^n^) parents in the progeny lines. In wheat, two MAGIC populations, with four and eight founders respectively,have been established [60]. Comparison of thefour-way MAGIC population with a consensus map incorporating over 100 bi-parental populations showed that several regions where markers could not be separated by recombination in the consensus map are spread out over 10 to 20 cM in the MAGIC map[60]. This increase in resolution within the four-way MAGIC population is expected to be even higher with a greater number of founders and therefore,the eight-way MAGIC population [60]represents an attractive solution for anchoring wheat physical maps in the future. Another possibility to increase recombination is to use linkage disequilibrium (LD) mapping. The extent of LD,or non-random association of alleles at linked loci, depends on the recombination rate, the mating system, the domestication process, the natural and artificial selection,and the population structure[61]. LD can persist over tens to hundreds of kilobases in selfing species like *Arabidopsis thaliana*[62] or soybean [63] and can decline over a few hundred base pairs in outcrossing species like maize [64]. In wheat, the LD extent was estimated between 300 and 500 kb (r^2 ^= 0.2) depending on the geographical origin of the population analyzed [65].LD mapping is developing rapidly in wheat through the construction of association panels[66-68]and the exponential increase in the number of markers,thereby offering new perspectives for anchoring physical maps in wheat.

The second strategy to overcome the lack of recombination in wheat is radiation hybrid mapping,which is completely independent from recombination as it relies on radiation-induced chromosome breakage and the reconstruction of the markers order based on co-retention analysis[69,70]. A pilot study during the construction of the 3B physical map indicated a resolution of 263 kb for the 3B radiation hybrid mapping panel [14] and, more recently, Kumar *et al.*[71]estimated a resolution <140 kb for a panel of the wheat D-genome progenitor *Aegilops tauschii*. Thus, radiation hybrid mapping provides a viable solution to resolving the order of physical maps in the two-thirds of the wheat chromosomes that show little or no recombination.

A few years ago, physical mapping in hexaploid wheat seemed out of reach. The success of the 3B and 1BL physical mapping projects and the many more physical maps to come in the near future through the coordinated efforts in the IWGSC [10] with theconstant improvement in methodologies opens new perspectives in wheat research. Wheat physical maps will gradually replace the aneuploid stocks that were used in the past decades for defining the location of markers and traits along wheat chromosomes. In addition,high resolution wheat physical maps will help to increase our understanding of factors underlying recombination[25], which in turn should enable the manipulation and improvementof recombination in pericentromeric regions.

### High density physical mapping provides new insights into wheat genome evolution

It is currently recognized that the common ancestor of grasses had, approximately 90 million years ago, five chromosomes, and underwent a whole genome duplication followed by inter-chromosomal translocations and fusions resulting in an intermediate ancestral genome (approximately 70 million years ago) with 12 chromosomes (A1 to A12) [29,56]. In wheat, chromosomes from groups 1 and 3 originate from chromosomes A5 and A1, respectively, which resulted from the whole duplication of proto-chromosome A5[40]. While ancestral wheat chromosome 3 evolved directly from chromosome A1 without major rearrangements, ancestral wheat chromosome 1resulted from the insertion of chromosome A10 into chromosome A5[40].As a consequence, only the distal 53% of chromosome 1BL share common ancestry with the long arm of chromosome 3B. In rice, which has retained the same 12 chromosomes structure as the intermediate ancestral genome, the chromosomes syntenic to wheat chromosome 3 and 1 are chromosomes 1 and 5, respectively. Murat *et al. *[41]identified 64 genes, still conserved in today's rice, *Brachypodium*,and sorghum genomesfrom the duplication of ancestral proto-chromosome A5.A similarity search between the 128 ancestrally duplicated rice genes and the Illumina contigs from the IWGSC survey sequencing of all wheat chromosomes identified 12 genes on wheat chromosomes 1BL and 3BL. When comparing the proportion of ancestral genes conserved from proto-chromosome A5 in wheat and rice, there wasabout four to eight times more retention in rice. This supports evidence that in wheat the ancestral gene backbone has been more rearranged than in rice[72,73] and indicates that wheat is not a good template for the reconstruction of the ancestral grass chromosome content. Among the 1BL gene set, 59.5% were identified as non-syntenic geneswith the other grass genomes confirming the hypothesis of active gene movements specific to the wheat lineage after divergence from the other grasses [18,28,73,74]. This ratio is very similar tothe estimates of Wicker *et al. *[28], who found 62.7% of non-syntenic genes using 454 sequencing of sorted chromosome 1BL. Here, access to the physical map enabled us to further analyze their distribution along the chromosome. The general pattern showed an increase in the proportion of non-syntenic genes from the centromere to the telomere, as previously observed in wheat [18,73-77]. However, apeak of synteny was observed in the proximal bin 1BL6-0.32-0-47. Interestingly, this bin corresponds to the distal end of the ancestral orthologous rice chromosome 10. The increase in the proportion of syntenic genes from the centromere to the telomeres has been observed systematically on the *Brachypodium*, sorghum and rice chromosomes [41],reflecting a general pattern for the distribution of syntenic genes along ancestral grass chromosomes. Therefore, the peak of synteny observed in the middle of chromosome 1B likely corresponds to the ancient telomeric synteny pattern whereas the increase of non-syntenic genes towards the telomere reflects the more recent history of the wheat genome evolution. Thus, we conclude that the synteny distribution along chromosome 1BL is the result of the superimposition of the ancestral grass and the recent wheat evolutionary patterns. Such apattern was not observed as clearly in a recent comparative study of *Ae. tauschii*, the diploid ancestor of the D genome of bread wheat [73]. In that study, the authors showed that the average synteny with both rice and sorghum chromosomes was significantly higher in the proximal half than in the distal half of the *Ae. tauschii *chromosome segments, reflecting the increase of the proportion of non-syntenic genes from the centromere to the telomere as seen on chromosome 1BL. However, they did not observe a significant difference in the synteny level between the proximal and the distal halves of chromosome 1DL that corresponds to the ancestral orthologous rice chromosome 10. This is likely due to the lower number of gene loci (21 versus 1,161) analyzed on chromosome 1DL compared to chromosome 1BL, illustrating the potential of high density physical mapping in comparative studies.

On chromosome 3B, detailed sequence analysis of 13 large contigs containing 175 genes distributed along the chromosome [18] and 2,924 genes anchored on the 3B physical map [15]indicated that 35% to 42% of the genes are not syntenic with other grass genomes. These results suggest a lower proportion of non-syntenic genes on chromosome 3B compared to chromosome 1BL (approximately 60%). Moreover, no peak of synteny was observed on wheat chromosomes 3BL and 3BS,in which there is a continuous increase in the proportion of non-syntenic genes along the chromosome arms towards the telomeres [15]. These differences aredue to the different evolutionary origin of homoeologous group 3 chromosomesin wheat, which, in contrast to chromosomes of group 1, originate directly from a single ancestral chromosome (proto-chromosomeA1)[78].Despite these differences, the density and proportion of gene islands,the gradient of gene density from the centromere to the telomere,and thecorrelation with the density of non-syntenic genes or the recombination rate remained very similar between chromosome 1BL andchromosome 3B [15].Thus, our results show that wheat chromosomes display a superimposition of evolutionary patterns. Some, such as the pattern of synteny, will differ between two chromosomes as a reflection of ancient history while others, such as the pattern of gene island formation and non-syntenic gene movement, will be similar because they reflect wheat lineage-specific and more recent history.

### The 1BL physical map: a landing padfor efficientmap-based cloning and sequencing

The size of the bread wheat genome (17 Gb) is a considerable challenge for map-based cloning. In particular, the large amount of repetitive DNA (>80%) and the presence of three homoeologous genomes (A, B and D genomes) represent major difficulties during chromosome walking. Consequently, only a few genes have been cloned in wheat so far[79]. In the absence of physical maps, comparative genomics has been used to supportmap-based cloning in wheat. The positional cloning of *VRN *genes on chromosomes 5A and 7BS [80-82], *Ph1 *on chromosome 5B [83], and *Gpc-B1 *on chromosome 6BS [84] in wheat benefited greatly from the available rice genome sequence. However, comparative analyses of wheat *Lr10*[85], *Lr21*[86] and *Pm3b*[87] disease-resistance genes with the rice genome sequence showed that it contains homologous genes to these three genes, but at non-orthologous positions, indicating that genomic rearrangement interruptscollinearity of wheat and rice in some genomic regions. We confirmed these interruptions between wheat and the other grass genomes for chromosome 1BL with a break of collinearity between deletion bins 1BL6-0.32-0.47 and 1BL1-0.47-0.61, and the high level of non-syntenic genes (59.5%). These three disease-resistance genes were isolated by positional cloning using diploid or tetraploid wheat genomes that are closely related to the genomes of modern hexaploid wheat as an alternative to the use of the rice genome sequence. However, this approach is very time-consuming and requires a variety of genomic resources. The low number of genes cloned in the wheat genome and more particularly on chromosome 1BL, for which none of the 40 QTLs mapped so far have yet been cloned, illustrates the current limits in positional cloning in wheat.

Here, we provide a powerful tool for map-based cloning on wheat chromosome 1BL with a high quality (93% of chromosome coverage, N50 = 1,128 kb) and very dense(11 markers per Mb) physical map including 1,161 genes, a good percentage of ordered contigs (48% of the chromosome arm) and a high level of anchoring (74% in the deletion bins and 19% in the genetic map). This provides potentially 916 markers including 193 genes to each of the 40 QTLs mapped on chromosome 1BL. With such information tohand, it is possible to define a subset of markers ordered along the interval and initiate fine mapping in a high resolution recombinant population to rapidly identify physical contigs that can provide additional markers and candidate genes or be sequenced. For nine QTLs mapped in the distal part of the chromosome arm (ratio of genetic to physical distance = 0.6 cM/Mb) with a confidence interval of less than 3 cM and an N50 value for the contig size of 1.1 Mb, one to three contigs should be sufficient to cover such intervals,providing landing pads for a rapid identification of potential candidate genes.

The physical map of wheat chromosome 1BL provided here was built with a BAC library constructed from the genotype Chinese Spring. However, in the final step of map-based cloning, it is often desirable to use a genomic library of the cultivar that contains the gene of interest. This problem was revealed in wheat by the analysis of the *Lr10 *gene locus. At this locus, two haplotypes thatwere defined by the presence (H1) or absence (H2) of two resistance-gene analogs were found in a collection of 113 wild and cultivated wheat lines [88]. The isolation of *Lr10 *was only possible because the BAC library used in the sub-genome map-based cloning approach was constructed from a genotype belonging to the H1 haplotype [85]. Thus, in some cases there will be a need to construct a new BAC library in another genotype than Chinese Spring. Here, the high density of markers anchored to the 1BLphysical map (11 markers per Mb)combined with an adapted pooling strategy of the new BAC library [89] should allow the rapid identification of BAC clones spanning the target region and chromosome landing.

The IWGSC has established a road map for obtaining a high quality reference genome sequence [90] ofthe hexaploid bread wheat genome following a chromosome-based strategy [11] to overcome the difficulties associated with the high level of ploidy.Only a high quality assembly allows completegenome information to be captured accurately, in particular the information embedded in the repetitive fraction, which has been shown to play key roles in evolutionary changes and regulatory innovation. This is of crucial concern for the wheat genome as it is primarily composed of repetitive elements. In this regard, the high quality physical map of wheat chromosome 1BL provides a robust platform for sequencing the 1BL chromosome using a BAC-by-BACapproach and adapted next-generation sequencing technologies.

## Conclusions

Using a combination of efficient assembly tools and high throughput genotyping platforms, we developed a high quality physical map representing 94% of wheat chromosome 1BL. The map is anchored (74%) and ordered (48%) with 5,489 markers, representing the highest density of markers (11 markers per Mb) so far for a wheat physical map.This provides a powerful tool for map-based cloning and a robust platform for sequencing the 1BL chromosome in a BAC-by-BAC approach.The high density of genes mapped on the 1BL physical map allowed us to gain new insights into the gene space organization.Further, it revealedthat the pattern of synteny along chromosome 1BL is the result of the superimposition of the ancestral grass and recent wheat evolutionary patterns.

## Materials and methods

### BAC Fingerprinting and data processing

A chromosome 1BL-specific BAC library named TaaCsp1BLhAcontaining 92,160 clones originating from sorted 1BL wheat chromosome of Chinese Spring was constructed as described by Simkova *et al. *[91]. Fingerprinting of all 1BL BAC clones was performed as described in Paux *et al. *[14]. Briefly, it consisted in the digestion of BAC DNA by five restriction enzymes (*Bam*HI, *Eco*RI, *Xba*I, *Xho*I and *Hae*III); labeling of the DNA fragments with the SNaPshot™ Multiplex Labeling Kit solution (Applied Biosystems, Foster City, CA, USA); and estimation offragment sizes on an ABI 3730XL DNA capillary sequencer (Applied Biosystems).

Data were processed using the GeneMapper, FingerPrint Background removal (FPB) and GenoProfiler programs to size the fragments and remove background noise and contaminations. In particular, raw electropherograms produced by the ABI Data Collection software were analyzed using GeneMapper: fragment sizing was performed without the 250base pair (bp) band of the 500LIZ GeneScan size standard file. Peak areas, peak heights and fragment sizes of each BAC fingerprint profile were exported in text format. Spurious peaks (background noise, vector bands, partial or unspecific digestions) and bands out of the range of 50 to 500 bp were removed by FPB; this software was also used to discard substandard profiles that may negatively affect contig assembly and to convert data into a format compatible with the GenoProfiler and FPC programs. GenoProfiler was used to detect cross-contaminated clones in 384-well and 96-well plates and to remove negative controls.

At the end, a total of 65,413 highquality fingerprints (71%) were obtained with an average number of scored bands per BAC fingerprint of 107 ±25 (ranging from 30 to 216). With an average insert size of 129 ±29 kb, the total size coverage of the fingerprinted BACs was 8,424 Mb.

### BAC assembly

Two software packages were used to build the physical map using the 65,413 high quality fingerprints: FPC and LTC. With the FPC software, automated assemblies were performed using the methodology described by Paux *et al. *[14] for the construction of the physical map of chromosome 3B. Briefly, the initial build of chromosome 1BL was performed by incremental contig building with a cut-off of 1e^-75^. These were subsequently run through single-to-end merging (Match: 1) at six successively higher cut-offs ending at 1e^-45^. The DQer function was used at each cut-off to break up all contigs that contained more than 10% of Questionable (Q) clones (Step: 3). The following parameters were used to establish the FPC physical map: a tolerance of 12, a gel length of 56,000 and a From End value of 55.

With the LTC software, the automated assemblies were performed using the following methodology: the same metric used with FPC, called the Sulston score, was used to calculate clone overlaps. Numerous groups of overlapping clones, called net of significant overlaps, were obtained with a cut-off of 1e^-15^. Then, subnetswere obtained at a cut-off of 1e^-25 ^and used to build contigs. All contigs with 5 to 999 clones and with linear topology (net width ≤1) were validated. All contigs with a width ≥2 were checked manually for their linearity. If only one clone explained the non-linearity, the contigs were validated because this non-linearity was likely due to the bad quality of the fingerprint for this clone. Then, all contigs with more than 999 clones and all non-linear contigs were broken up by the elimination of Q clones and Q overlaps. The same featuresas described previously (linearity and number of clones) were used to validate contigs. The remaining contigs were broken up by increasing the stringency (cut-off of 1e^-30^) and then elimination of Q clones and Q overlaps for the non-validated contigs. The following parameters were used to establish the LTC physical map: a tolerance of 12, a gel length of 60,000,and a minimum contig size of five clones.A manual version of the LTC physical map constructionwas then performed by identifying fingerprint overlaps with a lower stringency (cut-off of 1e^-15^), supported by information provided by contig anchoring in deletion bins with molecular markers as described in Paux *et al. *[14]. A home-made Perl script called FingMergingContigs.pl was developed to automate the identification of the contigs that need to be manually merged.LTC does not provide a consensus band map, which is needed to assign coordinates to the clones based on their alignment to the map [21]and allow theeasy ordering of markers inside physical contigs. Thus,to provide a gene order on the LTC physical map, the 616 LTC contigs were rebuilt with the FPC software to produce a consensus band map for each LTC contig.

### Minimal tilling path design and 3-dimentional pooling

The MTP was selected from the FPC automated physical map using the FPC software. The following parameters were used: a minimum FPC overlap of 30, a maximum FPC overlap of 250, a From End of 0 and minimum shared bands of 12.The three-dimensional pooling of the 8,597 clones of the MTP selected from the 1BL BAC library was performed as described in Paux *et al. *[14]. Itresulted in 24 column pools, 16 raw-pools, 23 plate pools, and a super pool containing all 8,597 BACs clones.

### Marker development

DNA amplified from 1BL sorted chromosomes was used for 454 shotgun sequencing as described by Wicker *et al. *[28]. A total of 2,177,887 good quality reads were obtained with an average read size of 383 bp and a total size of 834 Mb corresponding to a coverage of 1.6 X. Two home-made Perl scripts, IsbpFinder.pl and ssrFinder.pl, were used to develop ISBP and SSR markers, respectively, from the 454 reads. In total, 775,995 ISBPs and 38,400 SSRswere designed. Other home-made Perl Scripts were developed to automatically discard duplicated markers and select the best quality markers. The quality criteriawere the level of confidence given by IsbpFinder.pl [29] for the ISBP markers and more than 10 repeats for dinucleotide repeats for the SSR markers. Finally, 46,194 ISBP and 412 SSR corresponding to a total of 46,606good quality and non-redundant markers were identified.

### Plant material and DNA extraction

The specificity of the markers for chromosome1BL and marker assignment by deletion bin mapping was performed with aneuploid lines of Chinese Spring corresponding to: a nullisomic 1B-tetrasomic line, a ditelosomic 1BL line, a ditelosomic 1BS line [31,32], and eight deletion bin lines (1BL11-0.23, 1BL6-0.32, 1BL1-0.47, 1BL14-0.61, 1BL2-0.69, 1BL8-0.74, 1BL3-0.85 and 1BL4-0.89) [35].

The reference genetic mapping population was the same as the one described by Saintenac *et al. *[25] and was derived from the cross between cultivars Chinese Spring and Renan. F1 plants were self-fertilized and approximately 1,300 F2 seeds were sown to produce a single seed descent population. Among these lines, a set of 381 was used for genetic mapping. For each F2, 10F3 seeds were sown and leaves were harvested at a three-leaf stage for DNA extraction according the procedure described in Saintenac *et al. *[25].

### Genetic mapping

A total of 84 molecular markers (48 SSRs and 36 ISBPs) previously assigned to chromosome arm 1BL exhibited polymorphism between Chinese Spring and Renan and were selected for linkage analysis on the crossed population. The genetic map was constructed based on the maximum likelihood method using Mapmaker software [92] with a log of odds of 3 and θ of 0.25, applying the Kosambi [93] mapping function to transform recombination fractions into cM.

The chromosome 1BL neighbor map was constructed following the same strategy as for chromosome 3B [14]. Briefly, the Chinese Spring ×Renan genetic map from chromosome 1BL was used as a framework on which the position of loci mapped in another population was extrapolated. Loci shared between two maps were identified and used to define genetic intervals in which loci not present on the framework map (hereinafter referred to as 'target loci') were listed. Then, distances between shared and target loci were calculated as a ratio of the distance of the genetic interval and used, ultimately, to estimate the coordinate of the target loci on the neighbor map. The neighbor map was constructed with segregating data from the following mapping populations: Chinese Spring × Renanas a framework; W7984 × Opata (the ITMI reference population; GrainGenes[27]); RL4452 × AC Domain,SC8021-V2 × AC Karma,Wuhan × Nyubai (three populations from Agriculture Canada integrated in a consensus map including the ITMI map; Somers *et al. *[50]; Banks *et al. *[49]); Courtot × Chinese Spring[48]; and a wheat composite map originating from several tens of populations (R. Appels, personal communication).

### PCR amplification

PCR amplification was carried out in 10 µL reaction volume containing 5 µL of AmpliTaq gold 360 master mix (Applied Biosystems), 0.4 µL 360 GC enhancer (Applied Biosystems), 1 µL syto9 (2 µM), 1.6 µL forward and reverse primer (3 µM) and 2µL of 1/200 phi29 MTP pool amplification. PCR conditions were as follows: initial denaturation at 95°C for 10 min followed by 47 cycles of denaturation at 95°C for 30 s, annealing at 62°C for 30 s, extension at 72°C for 30 s, and a final extension at 72°C for 5 min. The PCR plates were then run on LightCycler 480 (Roche Diagnostics, Meylan, France{) to obtain dissociation curves. Results were then analyzed on LightCycler 480 Software release 1.5.0.

### NimbleGenarray development

High confidence ISBP markers were selected from the 46,194 ISBPsdesigned in the section 'markers development'. A mathematically defined repeats index using the Talymer program [94]and a Perl-based script were used to select specifically ISBP markers comprising a junction between a TE and a stretch of 30mer of low-copy sequence.Selected were 17,788 ISBP markers with an average of five probes per marker (88,470 probes), 27 positive controls (TE from wheat, 211 probes) and 20 negative controls (TE from several other species; 159 probes). Probes were 50 to 58 nucleotides in length (30mers for the low-copy DNA and a varying length of 20 to 28 nucleotides for the TE), with a temperature of melting (Tm) between 68°C and 73°C (Tm = 41 * ((nG + mC) - 16.4)/L + 64.9, where L = length of the oligonucleotide), and a GC content between 40% and 60%. All probes were oriented 5' LowCopyDNA_TE 3'(3' end being adjacent to the array surface). Selected probes were sent to design at Roche NimbleGen. All probes were randomly synthesized and spotted in a high density custom NimbleGen 12x135k array(Roche NimbleGen, Inc.).

### Microarray hybridizations

For the 63 1BL chromosome MTP BAC pools,500 ng of DNA was labeled using the NimbleGen Dual color labeling kit (Roche NimbleGen Inc.) according to the manufacturer's protocol. Dual color hybridizations were performed on each plex of the arrays.For the 1BL sorted chromosomes, 300 ng of DNA was labeled using the same kit. A dye swap was performed for this sample.

An updated version [A-MEXP-2314] of thewheat NimbleGen 40k unigene microarray[15], was hybridized according to the manufacturer's protocol with an additional vortexing step ofthe hybridization solution master mix (chapter 4 steps 1 to 4 of the protocol for hybridization and washing). Hybridization was performed for 72 hours at 42°C instead of 24 hours as in the original protocol. Washing steps were performed according to the manufacturer's protocol for gene expression analysis (Roche NimbleGen Inc.)

Hybridization and washing of the NimbleGen 17k 1BL ISBP arraywere performed according to manufacturer's procedure except that the hybridization time was extended to 120 hours. The arrays were dried by centrifugation at 1,200 × g for 1 min.

The arrays were scanned using the InnoScan 900AL scanner (Innopsys, Carbonne, France). Data were extracted from scanned images using the NimbleScan 2.5 Software (Roche NimbleGen Inc.) that allows for automated grid alignment, extraction and generation of data files.

### Microarray hybridization results analysis

For the wheat NimbleGen 40k unigene microarray,the normalization of the MTP pool data was done using the methods developed by Rustenholz *et al. *[15,16]. Two thresholds were calculated:the 'mean + × x Standard Deviation' with the following coefficient for the plates, columns and rows:plate: 2.7, 2.6, 2.5, 2.4, 2.3, 2.2, 2.1 and 2; row: 2.8, 2.7, 2.6, 2.5, 2.4, 2.3 2.2 and 2.1; column: 3.0, 2.9, 2.8, 2.7, 2.6, 2.5, 2.4, 2.3 and 2.2, andthe 't-test' method using the same thresholds as Rustenholz *et al. *[16]. All probes with a probe signal above these thresholds were considered positive.

The normalization of the sorted 1BL chromosome data was done using automated script developed with the R software[95]. A lowess correction was used to correct the dye biases. Afterwards the corrected intensity values were checked for each gene and the aberrant values deleted. Then, for each value, the median value of all genes was subtracted and divided by their standard deviation. Finally, the positive genes were identified with the 'mean + × x Standard Deviation' as described above.

For the 1BL ISBP NimbleGen array,the normalization was performed using automated scripts developed with the R software. The background intensity was estimated using the median of the intensities of the empty spots and subtracted fromthe intensity of each spot. After a log2 transformation, linear-lowess normalization was performed. Data were then subtracted by the median of the total spot intensity and divided by the standard deviation. For each probe,two thresholdswere calculated:the mean + 2 × the standard deviation and a Student's t-test at a *P*-value threshold of 0.05 were performed. All markers with at least 75% of their probes above this threshold and with a *P*-value below 0.05 were considered positive for a pool.

### Three-dimensional MTP pools and plate pools data deconvolution

A home-made Perl script, called DSMP.pl was developed for the deconvolution of molecular marker screening data produced on the three-dimensionalBAC pools of the MTP. Essentially, the script checks if two overlapping BACs of the physical map explain all the positive pools and can alsodeconvolute three others types of results: two non-overlapping BACs from two different contigs with fingerprints matching at acut-off of 1e^-25^, a tolerance of 12, and a MTP addresses that explain all the true positive pools; two overlapping BACs with one fingerprint matching a third BAC, included in a contig other than the two overlapping BACs, at a cut-off of 1e^-25^, a tolerance of 12, and a MTP addressing these three BACs that explain all the real positive pools;and a pair of overlapping BACs with one fingerprint matching a BAC in a second pair of overlapping BACs, included in a contig other than the two first overlapping BACs, at a cut-off of 1e^-25^, a tolerance of 12, and a MTP addressing these fourBACs that explain all the true positive pools. TheDSMP.pl script is available upon request from the corresponding author.

The molecular marker screening data produced on the plate pools of the whole 1BL BAC library were deconvoluted with the Elephantsoftware and the default parameters[33].Briefly, Elephantpartitions the contigs into short sections by splitting the contig at each branching point and establishes a list of clones for each segment; for each marker, it combines the results from pool screening with the pool composition to establish a list of candidate clones harboring the markers; it compares the two lists and scores each segment; and finally, for each marker, if a unique segment had a score above the threshold of 13, it assigns the marker to the segment.

### Roche454 sequence information

The Roche454 sequencing of the 1BL sorted chromosome was performed by Wicker *et al. *[28]. They produced 2,177,887 reads and covered 834 Mb (1.6 X). All sequence information generated was deposited to the European Bioinformatics Institute short-read archive under the accession number [ERX009439].

### Sequence analysis

Sequences were analyzed using Basic Local Alignment Search Tool (BLAST) software [96]. BLASTN analyses of all the probes of the wheat NimbleGen 40k unigene microarray [15] against all the Roche454 reads of sorted chromosome1BL were performed to identify the 1BL unigenes present on the NimbleGen microarray. The results were parsed to keep the best hit with at least 98% of sequence identity on at least 57 bp. Every unigene with a hit meeting these criteria wasconsidered as aunigene originating from chromosome 1BL.

BLASTXanalyses of the 39,179 unigenes sequences represented on the wheat NimbleGen 40k microarray against the databases of all rice (*Oryza sativa*) [97], *B.distachyon*[98] and sorghum (*Sorghum bicolour*) peptides [99] were performed to identify orthologous genes to the genes mapped on wheat chromosome 1BL. The results were parsed to keep the best hits with at least 35% of sequence identity on at least 40 amino acids. Every unigene with a hit meeting these criteria was considered as orthologous of the rice, sorghum or *B. distachyon *gene identified.

To eliminate redundancy in the unigene set, we used information from orthologous genes in rice and *B.distachyon*. We also checked with the latest release of the wheat unigene build (version 59) for further redundancy compared to the build version 55 that was used to build the wheat NimbleGen 40K unigene microarray[15]. When unigenes were located on the same BAC(s), had the same orthologous genes in rice and/or in *B.distachyon*, and were grouped in the same cluster of the new build version 59, only one unigene was kept on the 1BL physical map.

TBLASTXanalyses of the coding region of 128 rice genes corresponding to 64 ancient duplicated genes between rice chromosome 1 and 5 as defined by Murat*et al. *[41]were performed against all the sequence contigs from the IWGSC survey sequencing of all wheat chromosome. The results were parsed to keep the cumulated hits with at least 35% of sequence identity on at least 70% of the coding region of the rice genes.

### 1BL GenomeZipper construction

The GenomeZipper [36,37] of chromosome 1BL was builtusing a comparative framework with reference grass genomes and 242 gene-based single nucleotide polymorphism markers from thosemapped by genotyping by sequencing on chromosome 1BL[38]. To identify and position genic regions covered by the wheat 1BL sequence contigs (198,968 contigs) produced by the IWGSC [10], repetitive elements were detected and maskedby comparingthe wheat 1BL assembled sequences against the MIPS-REdat Poaceae v8.6.2 repeat reference library using Vmatch [100]and the following parameters: 70% identity cut-off, 100 bp minimal length, seed length 14, exdrop 5, and e-value 0.001. These sequences were then sequence masked and not considered for the construction of the genome zipper.

To identify syntenic conserved genes in the repeat-filtered wheat 1BL sequence contigs, sequence comparisons (BLASTX, ≥75%/70% sequence identity, alignment length ≥30 amino acids) against three reference genomes of *B.distachyon *(genome annotation v1.2,[101]), rice (rice RAP-DB genome build 4[97]) and sorghum (genome annotation v1.4, [99]) were performed. Syntenic regions corresponding to wheat 1BL were calculated usinga sliding window approach (windows size 0.5 Mbp, shift size 0.1 Mbp) to define synteny by the density of homology matches between query and reference genome.

The corresponding orthologous genes were anchored to the marker backbone via bi-directional blast hits. Genes without marker association were located according to their position in the corresponding reference genomes. The obtained 1BL linear ordered gene map was then expanded using 1BL repeat filtered contigs, wheat ESTs ( v1.19) [39], and wheat full-length cDNAs [102].

### Gene ordering on chromosome 1BL

The ordering of genes along chromosome 1BL was based first on the ordering of the physical contigs and second on the position of the genes within contigs. Each 1BL gene was assigned to one or several overlapping BACs using the information from the 40k unigenes NimbleGen array.The position of each gene in each contig was estimated based on the average of the consensus band map coordinate of the BAC(s) containing the gene. When several genes were assigned to the same BAC(s), the order was based on the synteny information when available or randomly chosen if not. The position of the genes with a clear position relative to their neighbor or with synteny information was consideredto have high confidence. The position of the genes with unclear placement with their neighbor genes or with discrepancy between synteny information and position in the contig was consideredto have low confidence.

### Comparison of the 1BL virtual gene orders based on the physical map and the GenomeZipper

To compare the two virtual gene orders, we identified the common genes. BLASTN [96] analyses of all the 1,161 1BL unigenes against all the 1BL Roche454 reads and ESTs of the GenomeZipper were performed. All genes with at least 90% of sequence identity on at least 100 bp were considered as common genes. Moreover, we compared the orthologous genes identified in rice, sorghum and *B. distachyon *for the GenomeZipper and the 1,161 1BL unigenes andall genes with the same orthologous genes in one or more of the three cereals were considered as common genes.Figure 3D was drawn using Circos [103] to compare the order of the common genes between the virtual gene orders based on the physical map andthe GenomeZipper.

### Data availability

A genome browser of the physical map of the wheat chromosome 1BL is available from the Unité de Recherche Génomique Info website [34]. All the NimbleGen array design and all microarray data have been deposited to ArrayExpress [104] under accession numbers [A-MEXP-2314]for the wheat NimbleGen 40k unigene design, [A-MEXP-2312] for the 1BL ISBP NimbleGen array design and [E-MTAB-1657] for the ISBP hybridization experiment and [E-MTAB-1650]for the wheat NimbleGen 40k unigene hybridization experiment. The Roche454 sequences of the 1BL sorted chromosome areaccessible at the European Bioinformatics Institute short-read archive under the accession number [ERX009439].

## Abbreviations

BAC: bacterial artificial chromosome; BLAST: Basic Local Alignment Search Tool; bp: base pair; cM: centimorgan; COS: conserved orthologous set; Elephant: electronic physical map anchoring tool; EST: expressed sequence tag; FPB: FingerPrint Background removal; FPC: FingerPrinted Contigs; Gb: gigabase; kb: kilobase; ISBP: insertion site-based polymorphism; ITMI: International Triticeae Mapping Initiative; IWGSC: International Wheat Genome Sequencing Consortium; LD: linkage disequilibrium; LTC: Linear Topological Contig; MAGIC: multi-parent advanced generation inter-cross; Mb: megabase; MTP: minimal tiling path; QTL:quantitative trait loci; RFLP: restriction fragment length polymorphisms; SSR: single sequence repeats; TE: transposable elements; WGP: Whole Genome Profiling; WGS: whole genome shotgun.

## Competing interests

The authors declare that they have no competing interests.

## Authors' contributions

RP carried out the physical mapping, the anchoring of the physical map in the deletion bin and genetic maps, the comparative genomic analysis, the gene space analysis, and drafted the manuscript. EP supervised the physical mapping and the anchoring. IB produced all the PCR marker data and the hybridization on the wheat NimbleGen 40k unigene microarray and analyzed the hybridization results. PS produced all the genetic maps. FC participated in the development of the DMSP.pl Perl script for anchoring. CL developed the 1BL NimbleGen 17K ISBP microarray, produced the hybridization data and analyzed the hybridization results. HŠ, JS and JD sorted the DNA of the wheat chromosome 1BL and produced the 1BL BAC library. AB, SV and HB handled the 1BL BAC library and produced the three-dimensionalMTP pools. ZF and AK participated in the assembly of the physical map with LTC. FC, FM and SS produced the high-information-content fingerprints of the whole 1BL BAC library. MM and KM produced the GenomeZipper of the wheat chromosome 1BL and participated in the comparison of the two virtual gene orders. CF acquired the funding, supervised all the analyses and drafted the manuscript.

## Supplementary Material

Additional file 1 **Information on the 5,489 markers assigned to the physical map of wheat chromosome 1BL**. Table listing the marker name, the marker type, the names of the deletion bin,the ID of the BAC clones andthe name of the physical contigsto which the marker is assigned,the position (in cM) of the markerson the Chinese Spring ×RenanF2 genetic map,the order of the geneand the level of confidence in this order, the unigene length, the ID of the orthologous genes in rice, sorghum and *Brachypodium *and the gene islands number for the 1,161 genes assigned to the physical map of the wheat chromosome 1BL. Click here for file

Additional file 2 **Features of the 616 contigs of the final LTC physical map of the wheat chromosome 1BL**. Table listing the contig name, the number of clones, the contig size, the total number of markers, the number of genes, the name of the deletion bin to which the contig is assigned, the position on the 1BL neighbor genetic map and the number of the ordered contigs. Click here for file

Additional file 3 **GenomeZipper of wheat chromosome 1BL**. Table listing the marker name, the position on the genotyping by sequencing genetic map, the ID of the orthologous genes in rice, sorghum and *Brachypodium*, the ID of the full-length cDNA matching the marker or the orthologous genes, the wheat Illumina reads matching the marker or orthologous gene and the ID of the wheat ESTs matching the marker or orthologous gene. Click here for file
